# Evaluation of health-related quality of life after total hip arthroplasty: a case-control study in the Iranian population

**DOI:** 10.1186/s12891-019-2428-0

**Published:** 2019-01-31

**Authors:** Mansour Bahardoust, Mikaiel Hajializade, Reza Amiri, Fatemeh Mousazadeh, Karim Pisoudeh

**Affiliations:** 1 Department of Epidemiology, School of Public Health, Iran University of Medical Sciences, Tehran, Iran; 2 M. D, Resident of Orthopedic, Rasoul Akram Hospital, Iran University of Medical Sciences, Tehran, Iran; 3 MSc Student of Education Management, Faculty of Psychology and Educational Sciences, Islamic Azad University, South Tehran Branch, Tehran, Iran; 4 Bone and Joint Reconstruction Research Center, Shafa Orthopedic Hospital, Iran University of Medical Sciences, Tehran, District 12, Mojahedin Islam St, Tehran, IR Iran

**Keywords:** Total hip arthroplasty, Health-related quality of life, Iranian population

## Abstract

**Background:**

As the total hip arthroplasty (THA) mainly aims to improve the quality of life of the patients, study of health-related quality of life (HRQoL) after THA has attracted much attention. Yet, the results considerably vary between studies. Here, we evaluate the HRQoL of the patient after THA, for the first time in the Iranian population.

**Methods:**

In a case-control study, HRQoL was assessed in 217 patients after THA and compared with a matched reference population. The 36-item short-form health survey (SF-36) was used for the evaluation of HRQoL. A multiple linear regression model was used to investigate the influence of sociodemographic and clinical characteristics of the patients on the HRQOL.

**Results:**

The mean follow-up of the patients was 27 ± 18 months. The mean total SF-36 score was 41.4 ± 22.2 in the case and 67.3 ± 26.6 in the control group (*p* = 0.001). The mean physical component score, but not the mental component score, was significantly lower in the patient group (*p* = 0.001). Except for the vitality and emotional role, all other SF-36 subscales were significantly lower in the case group. Male sex (B = 4.52, *p* = 0.023), number of comorbidities (B = − 4.82, *p* = 0.011), body mass index (B = − 1.18, *p* = 0.044), number of post-operative complications (B = − 6.57, *p* = 0.001), and adherence to physiotherapy protocol (B = 2.09, *p* = 0.014) were associated with HRQoL after THA.

**Conclusion:**

Although THA is considered as one of the most successful orthopedic practices, it is associated with remarkable reduced HRQoL in Iranian population when compared with the reference population. A variety of patients-associated factors influence the HRQoL after THA.

**Electronic supplementary material:**

The online version of this article (10.1186/s12891-019-2428-0) contains supplementary material, which is available to authorized users.

## Background

Total hip arthroplasty (THA) is one of the most frequent and successful orthopedic surgeries that has been marked as the “operation of the century” [1]. According to the report of Pivec et al. [2] in 2012, more than one million THA surgeries are being performed every year worldwide, and as the population ages, this number is estimated to double within the next decade [2].

In spite of the remarkable improvement in pain, mobility, and physical function following the THA and considering a 10-year implant survival of greater than 95% and a 25-year implant survival exceeding 80% [3–5], not all the patients are satisfied with the result of THA. Several post-operative complications have been associated with THA, hereby reducing the quality of life of the patients. Wound complications, thromboemboli, neurovascular injuries, prosthetic dislocation/instability, implant loosening, periprosthetic fracture, limb-length discrepancies, abductor muscle disruption, periprosthetic joint infection, heterotopic ossification, osteolysis, cup-liner dissociation, and prosthesis fracture are included in the list of reported complications, leading to reoperation or revision surgery in many cases [6, 7]. It should also be noted that it is not necessarily THA that is associated with remarkable reduced HRQoL, but the mostly sicker people that undergo this procedure.

With the growing number of individuals living with a hip prosthesis, the patients’ expectations after THA have also increased, which has led to an increased interest toward the identification of the predictors of quality of life following the THA. While conventionally clinical parameters have been used to assess the outcomes of patients after THA, a recent consensus states the patient-reported Health-related quality of life (HRQoL) is a better indicator of patients’ outcome and should be primarily used in due researches and clinical setting as well [8]. Accordingly, a wide range of studies has been devoted to the evaluation of HRQoL after THA [9]. Yet, the results of the HRQoL assessment after THA vary considerably between studies [9]. In addition to the patient-related factors such as age, gender, comorbidity, and preoperative functional status that have been associated with the results of HRQoL, the role of other factors such as individual orthopedic surgeon have also been noticed [10]. Thus, a thorough evaluation of HRQoL of patients after THA is still encouraged.

Here we aim to evaluate the HRQoL of patients after THA in comparison with a matched reference population and investigate the factors that might have predictive role in this scenario. To the best of our knowledge, this is the first large-scale study of this type in the Iranian population.

## Methods

**Fig. 1:**
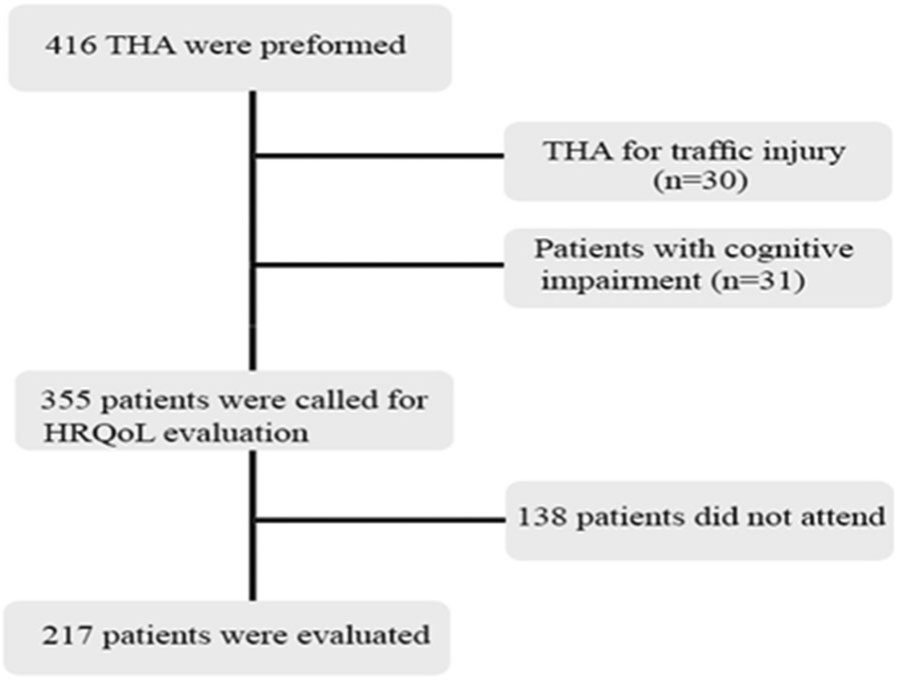
The flowchart of the study

This study was approved by the ethics committee of our institute and informed consent was obtained from participants before their inclusion in the study. In a case-control study, the patients who have undergone THA for a hip fracture or joint damage between September 2014 to September 2017, by either of the three orthopedic surgeons in the two Iranian university hospital (Shafa Yahyaeian and Rasul Akram hospitals), were identified. The inclusion criteria were the total hip replacement (femur neck/intertrochanteric) and availability for follow-up evaluations. The THAs that were performed to treat traffic injuries and those that were performed in patients with cognitive impairment were excluded. Patients with the follow-up of fewer than six months were excluded from the study as well. From a total of 416 patients who underwent THA during the study period, 61 patients were excluded as their THA was performed to treat traffic injuries (30 cases) or the patients had cognitive impairment (31 cases). Moreover, 138 patients did not attend the final evaluation session. Finally, 217 eligible patients were included in the study (Fig. 1).

The clinical and radiographic data of patients were extracted from their medical records and included the type of fracture (femoral neck/intertrochanteric), the time past the surgery, post-operative complications, the date of fist post-operative complication.

At the evaluation session, the quality of life questionnaire was filled by the patients. The short form health survey (Additional file 1) [11] was used to assess the quality of life, which its validity and reliability in Iran was confirmed by Montazeri et al. [12]. The questionnaire consisted of 36 questions in eight sub-scales (physical performance, physical role, physical pain, general health, vitality, social role, emotional role, and mental health). Each scale was directly converted into a 0–100 scale, where a lower score was equivalent to more disability vice versa. The total SF36 score and sub-scale SF36 scores were both reported. The physical component score (PCS) and mental component score (MCS) were also calculated using the norm-based scoring method. PCS is comprised of four subscales including physical function, role limitations caused by physical problems, bodily pain, and general health [13]. A matched reference population of 217 subjects with no history of hip fracture and/or THA was recruited from the community as the control group. Group matching was done based on the age, gender, and BMI of the patients. In case the patient or control subject was not able to complete the questionnaire, it was completed with the help of a researcher who was not involved in the analysis of the results.

The sociodemographic information including age, sex, body mass index (BMI), education, marital status, and smoking history was recorded. The number of comorbidities was also recorded in both the case and control group. In this respect, the presence of 14 comorbid conditions was investigated in the participants by asking questions. The questions corresponded to 12 general comorbid conditions (heart disorders, vascular disorders, hypertension, diabetes, kidney diseases, lung disorders, neurological problems, cancer, peptic ulcer, vision problems, low back pain, and psychiatric disease) and 2 musculoskeletal comorbidities (any regional pain other than hip pain and widespread pain). The number of comorbid conditions (0, 1, 2, or more) was compared between case and control group [14].

### Statistical analysis

SPSS for windows version 16 was used for statistical analysis of data. After the assessment of the distribution pattern of the variables, comparison of mean between the two study groups was done using parametric tests (independent t-test and analysis of variance) for normally distributed variables or their non-parametric counterparts (Mann–Whitney U test and Kruskal–Wallis test) for non-normally distributed variables. Chi-Square test was used to evaluate the relationship between the categorical variables. Multivariate analysis (linear regression) was used to examine the relationship between the two or more explanatory variables. A *p* value less than 0.05 was considered significant.

## Results

In total, 217 patients and 217 matched reference subjects were included in this study. The patients’ population included 147 (67.7%) females and 70 (32.3%) males with the mean age of 58.2 ± 16.1 years. The reason of THA was the elderly osteoarthritis in 121 (55.8%) patients, avascular necrosis in 45 (20.7%) patients, post-traumatic osteoarthritis in 32 (14.7) patients, and hip dysplasia in 19 (8.8%) patients. Fracture location was the femur in 165 (76%) patients and intertrochanteric fracture in 52 (24%) patients. The mean follow-up period of the patients was 27 ± 18 months. Ninety-nine (45.6%) patients completed the post-operative physiotherapy protocol. The control group consisted of 138 (63.6%) females and 79 (36.4%) males with the mean age of 58.4 ± 15.9 years. The mean BMI was 24.1 ± 3.1 kg/m^2^ in the case and 23.8 ± 3.8 in the control group. Hypertension, diabetes, and cardiovascular disorders were the most frequent comorbidities in both the case and control group. No statistical difference was found between the descriptive characteristics of the case and control group (Table 1).

**Table 1:** Comparison of demographic characteristics and number of comorbidities between the case and control group

Variable	Case group (*n* = 217)	Control group (*n* = 217)	*P* value*
Age (year)	58.2 ± 16.1	58.4 ± 15.9	0.4
Gender			
• Female	147 (67.7)	138 (63.6)	0.12
• Male	70 (32.3)	79 (36.4)	
Marriage status			
• Married	151 (69.6)	161 (74.2)	0.11
• Single	19 (8.7)	14 (6.4)	
• Divorced	9 (4.1)	10 (4.7)	
• Widowed	38 (17.6)	32 (14.7)	
Educational status			
• Illiterate	19 (8.7)	13 (6)	0.09
• Under diploma	44 (20.3)	50 (23)	
• Diploma	57 (26.3)	63 (29)	
• College education	97 (44.7)	91 (42)	
Smoking history			
• Positive	89 (41)	77 (35.5)	0.061
• Negative	128 (59)	140 (64.5)	
BMI (kg/m^2^)**	24.1 ± 3.1	23.8 ± 3.8	0.081
Number of comorbidities			
• 0	59 (27.2)	61 (28.2)	0.21
• 1	98 (45.1)	101 (46.5)	
• 2	46 (21.2)	43 (19.8)	
• > 2	14 (6.5)	12 (5.5)	

*Data are demonstrated as mean ± SD or number (%). *P* value of less than 0.05 is considered significant, **BMI body mass index

The mean overall SF-36 score was 41.4 ± 22.2 in the case and 67.3 ± 26.6 in the control group. This difference was statistically significant (*p* = 0.001). The mean PCS was 31.2 ± 9.7 in the case and 61.4 ± 11.8 in the control group (*p* = 0.001). The mean MCS was 51.6 ± 17.6 in the case and 52.1 ± 8.3 in the control group (*p* = 0.71). The most significant difference in the SF-36 subscales of patients and controls was observed in the physical performance, physical role, and social role. In this respect, the physical performance score was 28.3 ± 11.1 in patients and 71.6 ± 21.5 in the controls (*p* = 0.001). The physical role score was 31.2 ± 12.6 in patients and 73.4 ± 29.1 in controls (*p* = 0.001). The social role score was 28.5 ± 29.2 in the patients and 61.3 ± 32.6 in the controls (*p* = 0.001). All other SF-36 subscales scores, except for the vitality and emotional role score, were significantly lower in the patient group as well (Table 2).

**Table 2:** Comparison of the SF-36 total and sub-scales’ score between the case and the control group

Sub-scale	Case group (*n* = 217)	Control group (*n* = 2017)	*P* value*
Physical performance	28.3 ± 11.1	71.6 ± 21.5	0.001
Physical role	31.2 ± 12.6	73.4 ± 29.1	0.001
Mental health	39.3 ± 28.2	62.1 ± 31.4	0.014
Happiness and vitality	56.2 ± 21.4	68.7 ± 32.5	0.059
Emotional role	79.1 ± 19.2	77.7 ± 20.9	0.32
Social role	28.5 ± 29.2	61.3 ± 32.6	0.001
Physical pain	48.2 ± 28.4	70.1 ± 25	0.016
General health	42.1 ± 26.3	64.2 ± 26.7	0.023
Total score of quality of life	41.4 ± 22.2	67.3 ± 26.6	0.001

*A *p* value fewer than 0.05 is considered significant

In total, 35 complications were recorded in the case group following the THA, which included the deep infection (14 patients, 6.5%), dislocation (9 patients, 4.2%), prosthesis loosening (6 patients, 2.8%) and prosthesis fracture (2 patients 0.9%), periprosthetic fracture (2 patient, 0.9%) and neurovascular injuries (2 patient, 0.9%). The mean time period from the surgery to the first complication was 9.6 ± 7 months.

The mean total SF-36 score was 26.2 ± 9.82 in patients who had complications versus 46.4 ± 11.4 in patients without complications (*p* = 0.014). The mean total SF-36 score was also significantly higher in patients with the follow-up of > 9 months (48.61 ± 11.8) versus patients with the follow-up of < 0.9 months (37.7 ± 10.76) (*p* = 0.043). No significant difference was observed between the mean total SF-36 score of the patients who were treated for osteoarthritis versus those who had undergone THA for hip fracture (... Vs ..., *p* = 0.14).

In the multivariate analysis, postoperative complications were inversely associated with the total SF-36 scores of the patients (B = − 6.57, *p* < 0.001). Moreover, the total SF-36 was associated with the gender of patients, as females recorded a considerably lower score than males (B = 4.52, *p* = 0.023). The lower BMI of the patients was also associated with the better post-operative HRQoL (B = − 1.18, *p* = 0.044). Adherence to the post-operative pelvic physiotherapy protocol was associated with a better total SF-36 score (B = 2.09, *p* = 0.014) as well. In addition, number of comorbidities was associated with the HRQoL of the patients, as patients with higher number of comorbidities recorded worse SF-36 scores (B = − 4.82, *p* = 0.011). No other significant association was found between total SF-36 score and other descriptive characteristics of the patients including the age, follow-up period, educational/marital status, smoking history, and individual surgeon. Table 3 demonstrates the influence of sociodemographic and clinical characteristics of the patients on HRQoL in a multiple regression model.

**Table 3:** The multiple regression model showing the influence of patient-associated factors on health related quality of life of after total hip arthroplasty

Variable	Mean SF-36 score	B	Std. Error	t	*P*-value
Post-operative Complications					
• Positive	23.36 ± 10.7	−6.57	2.01	−2.54	0.001
• Negative	43.2 ± 11.3				
Adherence to pelvic physiotherapy					
• Yes	46.2 ± 12.1	2.09	1.46	1.81	0.014
• No	32.1 ± 9.8				
Gender					
• Female	32.1 ± 11.8	4.52	1.54	2.11	0.023
• Male	45.4 ± 13.2				
Marriage status					
• Married	42 .1 ± 11.4	2.78	2.06	1.34	0.41
• Single	39.2 ± 10.6				
• Divorced	39.6 ± 9.3				
• Widowed	38.9 ± 10.17				
Educational status					
• Illiterate	37.1 ± 11.5	3.41	2.44	1.68	0.38
• Under diploma	42.2 ± 12.7				
• Diploma	41.07 ± 12.4				
• College education	41.8 ± 11.2				
Smoking history					
• Positive	40.8 ± 10.9	−1.98	1.13	−1.26	0.23
• Negative	42.4 ± 11.7				
Surgeon number					
• 1	42.08 ± 12.1	2.54	2.17	1.88	0.6
• 2	40.3 ± 11.8				
• 3	38.6 ± 11.3				
Number of comorbidity					
• 0	45.7 ± 11.3	−4.82	1.97	−.1.56	0.011
• 1	43.9 ± 10.7				
• 2	34.8 ± 11				
• > 2	27.2 ± 12.4				
BMI (kg/m^2^)	–	−1.18	0.54	.42	0.044
Follow-up	–	1.76	1.08	.98	0.12
Age	–	1.48	.91	.84	0.78

## Discussion

While in the traditional orthopedic practice the patients’ perspective used to receive less attention than the clinicians’ prospective of impairment, recently there has been a shifting toward the patient-based measure of outcome [15]. Since THA mainly aims to improve the quality of life of the patients, assessment of the HRQoL on the evaluation of treatment outcome is now considered critical to fully understand the effects of this intervention [16].

In this study, we assessed the HRQoL in a large series of patients after THA and compared it with the HRQoL of a matched reference population as the control group. At a mean follow-up period of 27 months, the mean total SF-36 score of the patients was significantly lower in the case group in comparison with the reference group. The mean PCS was significantly lower in patients as well, while the MCS was not considerably different between the case and control group. Besides, all the SF36 sub-scales except vitality and emotional role were significantly lower in the patients in comparison with the reference population. According to our multivariate analysis, male gender, lower BMI, lower number of comorbidities and post-operative complications, and adherence to the physiotherapy protocol were positively associated with the total SF-36 score.

Considering the increasing worldwide incidence of THA [17, 18], many studies have focused on the evaluation of HRQoL of the patients after THA. Shan et al. in 2014 performed a systematic review and meta-analysis on the clinical studies published after January 2000 evaluating the mid-term HRQOL after THA in patients with osteoarthritis. A total of 20 studies were included in their study. According to their review, the mid-term post-operative HRQOL was superior when compared to the pre-operative measures on qualitative and quantitative analysis. Pooled response means of total Harris Hip Score (HHS), combined pain and physical function sub-scales of Western Ontario and McMaster Universities Osteoarthritis Index and HHS were also improved considerably up to 7 years. SF-36 analysis showed that physical functioning, bodily pain, physical role, emotional role, and social functioning were improved up to 7 years as well. Based on their results, the HRQOL was at least as good as reference populations in the early post-operative years, while subsequently plateaus or declines. They also reported a significant heterogeneity amongst the studies and urged further studies based on consistent guidelines [9]. In the current study, a comparison between pre- and post-operative HRQoL was not performed, as the study was designed in a retrospective approach. Yet, the HRQoL of the patients was significantly lower in patients compared to the matched reference population. It is of note that the majority of the studies included in the review of Shan et al. were performed in the developed countries, while here we reported the HRQoL of the patient in a developing country. Gordon et al. compared the HRQoL between the Swedish and Danish patients after THA. Based on their study, Danish patients had a significantly superior HRQoL than Swedish patients [19]. This result shows that HRQoL could be even considerably different between the two developed countries.

In one of the few studies performed in the developing countries, Shi et al. evaluated the HRQoL after THA is Taiwanese population. The SF-36 form was used for the evaluation of HRQOL in 131 patients who participated in the pre-operative and post-operative assessments. Post-operative assessments were performed at five time-points, including three months, six months, one year, two years, and five years after the THA. Based on their results, old age, female sex, a higher number of comorbidities, readmission within the last 30 days, and inferior preoperative function were negatively associated with post-operative HRQoL. They suggested that the patients should be informed that their post-operative quality of life depends not only on their post-operative care but also on their pre-operative functional status [10]. In contrast to the study of Shi et al., the systematic review of Santaguida et al. revealed that younger age and male sex were associated with the increased risk of revision 3- to 5-fold after THA [20]. While female sex and number of comorbidities were predictors of inferior HRQoL in our patients, we did not find any association between the age and the HRQoL of the patients. Considering the fact that female population of our study was nearly twice as the male population and as the females revealed an inferior HRQoL than males, the low HRQoL of the patients in this study could be justified at least in part. The BMI of the patients was also associated with the post-operative HRQoL and patients with lower BMI scores better in total SF-36. The predictive role of BMI on the HRQoL after THA has also been reported in other investigations [21, 22].

Our analysis revealed that post-operative care, specifically adherence to the physiotherapy protocol, is positively associated with the HRQoL and should be given more attention. Di Monaco et al. systematically reviewed the role of rehabilitation after THA. According to their results, post-operative physical exercise could be optimized with some additive early and late interventions, thereby improving the quality of life of the patients after the THA [23]. The study of Su et al. also revealed that early rehabilitation can remarkably reduce the incidence of postoperative complications such as prosthetic infection, and the number of outpatient visits within the first year after THA [24].

Our study had some weaknesses which should be pointed out. The main limitation of this study was its retrospective design that did not allow comparing the post-operative HRQoL of the patients with their pre-operative measures. Another limitation of this study was the relatively short follow-up of the patients. Thus further study removing these limitations is warranted in the Iranian THA population.

## Conclusion

Although THA is considered as one of the most successful orthopedic practices, the results of this study showed that the HRQoL of the patients after THA is considerably lower than the reference population. Factors such as gender and number of post-operative complications were found to have a predictive role in post-operative HRQoL that their impact might be difficult to control. Yet, weight loss and the improvement of post-operative rehabilitation through monitoring the patients’ adherence to physiotherapy could be suggested as an approach to improve the HRQoL of the patients, thereby reducing the post-THA health and financial burden for both the patients and the societies.

## Additional file

Additional file 1: SF-36 questionnaire. (PDF 200 kb)

## Abbreviations

BMI: Body Mass index
HHS: Harris Hip Score
HRQoL: Health-Related Quality of Life
SF-36: 36-tem short-form Health Survey_
THA: Total Hip Arthroplasty
