# Supplementary material for: Evaluation of health-related quality of life after total hip arthroplasty: a case-control study in the Iranian population
Source: BMC Musculoskelet Disord. 2019 Jan 31;20:46. doi: 10.1186/s12891-019-2428-0 (PMC6357390; doi:10.1186/s12891-019-2428-0)
Supplement: Supplementary file 1 — SF-36 questionnaire. (PDF 200 kb) [file 12891_2019_2428_MOESM1_ESM.pdf]

SF-36 QUESTIONNAIRE

Name:____________________ **Ref. Dr:___________________** **Date: _______**

ID#: _______________ **Age: _______** **Gender: M / F**

Please answer the 36 questions of the **Health Survey** completely, honestly, and without interruptions.

GENERAL HEALTH:
In general, would you say your health is:

- Excellent
- Very Good
- Good
- Fair
- Poor

Compared to one year ago, how would you rate your health in general now?

- Much better now than one year ago
- Somewhat better now than one year ago
- About the same
- Somewhat worse now than one year ago
- Much worse than one year ago

LIMITATIONS OF ACTIVITIES:
The following items are about activities you might do during a typical day. Does your health now limit you in these activities? If so, how much?

Vigorous activities, such as running, lifting heavy objects, participating in strenuous sports.

- Yes, Limited a lot
- Yes, Limited a Little
- No, Not Limited at all

Moderate activities, such as moving a table, pushing a vacuum cleaner, bowling, or playing golf

- Yes, Limited a Lot
- Yes, Limited a Little
- No, Not Limited at all

Lifting or carrying groceries

- Yes, Limited a Lot
- Yes, Limited a Little
- No, Not Limited at all

Climbing several flights of stairs

- Yes, Limited a Lot
- Yes, Limited a Little
- No, Not Limited at all

Climbing one flight of stairs

- Yes, Limited a Lot
- Yes, Limited a Little
- No, Not Limited at all

Bending, kneeling, or stooping

- Yes, Limited a Lot
- Yes, Limited a Little
- No, Not Limited at all

Walking more than a mile

- Yes, Limited a Lot
- Yes, Limited a Little
- No, Not Limited at all

Walking several blocks

- Yes, Limited a Lot
- Yes, Limited a Little
- No, Not Limited at all

Walking one block

- Yes, Limited a Lot
- Yes, Limited a Little
- No, Not Limited at all

Bathing or dressing yourself
◯Yes, Limited a Lot ◯Yes, Limited a Little ◯No, Not Limited at all

PHYSICAL HEALTH PROBLEMS:
During the past 4 weeks, have you had any of the following problems with your work or other regular daily activities as a result of your physical health?

Cut down the amount of time you spent on work or other activities
◯Yes ◯No

Accomplished less than you would like
◯Yes ◯No

Were limited in the kind of work or other activities
◯Yes ◯No

Had difficulty performing the work or other activities (for example, it took extra effort)
◯Yes ◯No

EMOTIONAL HEALTH PROBLEMS:
During the past 4 weeks, have you had any of the following problems with your work or other regular daily activities as a result of any emotional problems (such as feeling depressed or anxious)?

Cut down the amount of time you spent on work or other activities
◯Yes ◯No

Accomplished less than you would like
◯Yes ◯No

Didn't do work or other activities as carefully as usual
◯Yes ◯No

SOCIAL ACTIVITIES:
Emotional problems interfered with your normal social activities with family, friends, neighbors, or groups?

◯Not at all ◯Slightly ◯Moderately ◯Severe ◯Very Severe

PAIN:
How much bodily pain have you had during the past 4 weeks?

◯None ◯Very Mild ◯Mild ◯Moderate ◯Severe ◯Very Severe

During the past 4 weeks, how much did pain interfere with your normal work (including both work outside the home and housework)?

◯Not at all ◯A little bit ◯Moderately ◯Quite a bit ◯Extremely

ENERGY AND EMOTIONS:
These questions are about how you feel and how things have been with you during the last 4 weeks. For each question, please give the answer that comes closest to the way you have been feeling.

Did you feel full of pep?
- All of the time
- Most of the time
- A good Bit of the Time
- Some of the time
- A little bit of the time
- None of the Time

Have you been a very nervous person?
- All of the time
- Most of the time
- A good Bit of the Time
- Some of the time
- A little bit of the time
- None of the Time

Have you felt so down in the dumps that nothing could cheer you up?
- All of the time
- Most of the time
- A good Bit of the Time
- Some of the time
- A little bit of the time
- None of the Time

Have you felt calm and peaceful?
- All of the time
- Most of the time
- A good Bit of the Time
- Some of the time
- A little bit of the time
- None of the Time

Did you have a lot of energy?
- All of the time
- Most of the time
- A good Bit of the Time
- Some of the time
- A little bit of the time
- None of the Time

Have you felt downhearted and blue?

- All of the time
- Most of the time
- A good Bit of the Time
- Some of the time
- A little bit of the time
- None of the Time

Did you feel worn out?

- All of the time
- Most of the time
- A good Bit of the Time
- Some of the time
- A little bit of the time
- None of the Time

Have you been a happy person?

- All of the time
- Most of the time
- A good Bit of the Time
- Some of the time
- A little bit of the time
- None of the Time

Did you feel tired?

- All of the time
- Most of the time
- A good Bit of the Time
- Some of the time
- A little bit of the time
- None of the Time

SOCIAL ACTIVITIES:
During the past 4 weeks, how much of the time has your physical health or emotional problems interfered with your social activities (like visiting with friends, relatives, etc.)?

- All of the time
- Most of the time
- Some of the time
- A little bit of the time
- None of the Time

GENERAL HEALTH:
How true or false is each of the following statements for you?

I seem to get sick a little easier than other people
◯ Definitely true ◯ Mostly true ◯ Don't know ◯ Mostly false ◯ Definitely false

I am as healthy as anybody I know
◯ Definitely true ◯ Mostly true ◯ Don't know ◯ Mostly false ◯ Definitely false

I expect my health to get worse
◯ Definitely true ◯ Mostly true ◯ Don't know ◯ Mostly false ◯ Definitely false

My health is excellent
◯ Definitely true ◯ Mostly true ◯ Don't know ◯ Mostly false ◯ Definitely false
